# Using insulin pump with a remote-control system in young patients with diabetes improves glycemic control and enhances patient satisfaction

**DOI:** 10.1186/s40842-019-0081-z

**Published:** 2019-06-06

**Authors:** Asma Deeb, Mariette Akle, Layla Abdulrahman, Hana Suwaidi, Samar Awad, Sareea Remeithi

**Affiliations:** 1 Paediatric Endocrinology Department, Mafraq Hospital, Abu Dhabi, P O Box, 2951 United Arab Emirates; 2 Paediatric Endocrinology Department, Shaikh Khalifa Medical City, Abu Dhabi, UAE

**Keywords:** Insulin pump, Remote control, Satisfaction, Diabetes ketoacidosis

## Abstract

**Background:**

Insulin delivery triggered by a remote control is an advanced feature offered by newer insulin pump systems. These remote-integrated pump systems might further improve glycemic control and patient satisfaction. This study aims at assessing the effects of advanced insulin pump system on patients with type 1 diabetes mellitus (T1DM).

**Methods:**

The prospective, observational study in two centers addressed patients on multiple daily injection (MDI) switching to an integrated pump system (primary: adolescents and young adults, secondary: school-children). Treatment and patient satisfaction parameters were recorded at baseline and at two visits at 12 and 24 weeks.

**Results:**

Thirty-eight patients were analyzed; primary *n* = 24 (mean-age 16) and secondary *n* = 14 (mean-age 9). From baseline to visit2, the mean decrease of HbA1c was 1.09% (*p* = 0.00009) and 0.79% (*p* = 0.09) for the primary and secondary group, respectively. Patient satisfaction rate was favorable. Exploratory analyses revealed patients favoring the use of the remote control achieved best reductions in HbA1c (*p* = 0.0174). Safety was encouraging with no adverse events in the period from visit1 to visit2.

**Conclusions:**

Switching young T1DM patients from MDI to a remote control-integrated pump system achieved a reductions in HbA1c and insulin dose. Positive attitude towards remote operating enhanced these effects. Patient satisfaction has markedly improved.

**Electronic supplementary material:**

The online version of this article (10.1186/s40842-019-0081-z) contains supplementary material, which is available to authorized users.

## Introduction

It is well known that in the treatment of type 1 diabetes mellitus (T1DM), continuous subcutaneous insulin infusion (CSII) by pump systems results in marked advantages compared to multiple daily injections (MDI) [1–6]. Various studies in paediatric patients showed that there were improvements in HbA1c [7–11], along with lower glycemic variability [12], less complication events and improved long-term glycemic control [13–17]. In addition, multiple studies showed improved quality of life and treatment satisfaction for both patients and parents [18–20].

The advantages of CSII over MDI, probably, derive from two factors: firstly, the more physiological route of administration achieves better medical efficacy and secondly, the easier handling allows for improved the therapy adherence. Newer types of modern insulin pump have been used for treatment of T1DM. Integrated systems, utilizing incorporated multifunctional blood glucose meter and remotely controlled insulin pumps, were shown to be associated with a significant improvement of glycemic control without a change in patient satisfaction or quality of life [21]. Modern pumps with advanced features led to greater improvements in glycemic control [22]. Advanced pump functions may enhance patient satisfaction and improve glycemic control. For example, overall satisfaction with insulin pump system was further enhanced by a new sensitive occlusion detection mechanism that allow for earlier intervention [23]. A study by Picard et al. showed improved glycemic control and satisfaction with employing sensor augmented pumps [24].

The present study focused on advanced pump systems featuring a bolus calculator and insulin delivery triggered by a remote control. These systems have been used successfully in adults: In multicenter evaluations [25–27], these systems achieved improved glycemic control and treatment satisfaction for patients. For young patients, the compliance with T1DM therapy impacts a considerable burden, due to the perpetual interference with everyday activities in school, college, or workplace. We presumed that remote control for insulin delivery should ease adherence to therapy in view of special dress traditions common in some countries like the United Arab Emirates (UAE). Better therapy adherence bears the potential to further improve glycemic control and satisfaction compared to ordinary CSII systems.

The aim of the study is to assess the effects of employing an integrated pump system in adolescent patients or young adults (age 12–30). We also aimed to explore the suitability of the system for younger schoolchildren who may need assistance of parents or guardians also in operating the advanced features. By switching from MDI to an integrated system with remote control, we expected to see additional benefits seen for ordinary CSII systems. The observational design of the study does not allow to explicitly separate out the probable add-on benefits of the advanced features like the remote control. For getting a handle on this, additional question a specialized questionnaire (Additional file 1) was designed to measure the subjective usefulness of the advanced features and in particular of the remote control. These data were used in explorative analyses to assess the additional contribution of the remote control.

## Methods

### Study setup

The prospective observational study was approved by the research and ethics committee, Mafraq hospital and all data processing followed the laws of personal data protection. In two centers of the UAE, paediatric T1DM patients were enrolled who were willing to switch from MDI to an integrated system of pump therapy featuring a bolus calculator and insulin release via remote control (ACCU-CHEK® Combo, ACC, Roche Diabetes Care, for clinical evaluations see ([25–27]). Patients were trained on the pump use and the remote function. All patients were trained to a high level to use the technology and assessed to confirm that they have sufficient skills prior to switching to the new system.

An informed consent was signed by patients and/or parents/guardians. The primary study group included adolescent patients (age 12–17) and young adults (age 18–30) who are the main operator of the device. The secondary study group comprised schoolchildren aged 6–11 who may need assistance for operating the system by parents/guardians. Treatment parameters were recorded at baseline and in two follow-up visits at 12 and 24 weeks. Patient questionnaires dealing with the subjective aspects of therapy were used on baseline and on the two follow-up visits. The questions were grouped in 2 questionnaires to rate the treatment satisfaction on the new system (questionnaire A) and the technical side including the ease of handling, perceived therapy adherence and the usefulness of the remote control (questionnaire B). In addition, an analog scale was employed for measuring general satisfaction with treatment (Additional file 1). The questionnaires were developed by the study team and were validated by using in clinic for non-participating patients and clinic staff.

Standard practices of care were followed throughout the study.

### Statistical analysis

Statistical testing of all parameters and questionnaire results from baseline to visit 2 employed the non-parametric Wilcoxon signed-rank-test. Additional exploratory analyses addressed the effects on HbA1c under consideration of a potential contribution of advanced system features with focus on the remote control. For the latter, non-parametric Jonckheere-Terpstra trend test was employed along with parametric linear models.

### Power calculation

The intended size of the study (*n* = 40) provides sufficient power (85%) to detect a mean decrease in HbA1c of 0.5%.

## Results

Forty-three patients with T1DM were enrolled. Primary group *n* = 28 (mean age 16 ± 4.0 y, range 12–28) and secondary *n* = 15 (mean age 9 y ± 1.7 y, range 6–11). One Parent withdrew early and reverted to MDI as their 6-year-old child developed DKA, another patient returned to MDI after 3 months for personal preference. Three other patients terminated prematurely the study for reasons not related to therapy. Analysis data sets (complete HbA1c data for baseline and visit2) comprised 24 and 14 patients in the primary and secondary group, respectively. Table 1 summarizes descriptive data for the study populations at baseline. The majority of primary study group participants were girls (70.8%), school attendees (75%) and citizens of the UAE (75%). Mean HbA1c at baseline was 9.7% ± 1.7% (83 mmol/mol); mean duration of diabetes was 6.3 years ±5.1 y (for the secondary group, 8.7% ± 1.9%, 72 mmol/mol and 2.8 years ±2.0 y).

**Table 1:** Study populations at baseline

		Primary *N* = 24 Age 12–30 y		Secondary *N* = 14 Age 6–11 y	
	Variable	Mean	Std	Mean	Std
Demography	% FEMALE	70.8	–	64.3	–
	AGE [years]	16.0	4.0	9.0	1.7
	WEIGHT [kg]	52.9	11.7	31.6	12.2
	HEIGHT [cm]	156.1	8.3	130.3	12.6
	BMI [kg/m^2^]	21.5	4.1	18.0	3.6
	BMI PERCENTILE [%]	62.7	21.2	48.7	36.7
	ETHNICITY [% UAE]	75.0	–	42.9	–
Status	% married	4.2	–	0.0	–
	% school	75.0	–	100.0	–
	% college	16.7	–	0.0	–
	% employed	8.3	–	0.0	–
Diabetes	HbA1c [%] Mmol/mol	9.7 83	1.7	8.7 72	1.9
	DURATION DIABETES [years]	6.3	5.1	2.8	2.0

The change of parameters and questionnaire results from baseline over visit1 to visit2 for the primary study group are summarized in Table 2. There was a mean decrease of HbA1c 1.05% (*p* < 0.0001) from baseline to visit 2. In blood pressure and BMI there were no sizeable changes. There was a slight (6%) but significant decrease in average daily insulin (*p* = 0.03). Questionnaire results are reported in the lower part of Table 2. There was a significant increase in general treatment satisfaction, perceived handling ease, and subjective compliance.

**Table 2:** Parameters of the primary study group over the study period (*N* = 24 Age 12–30 y)

	Baseline		Visit 1		Visit 2		
	Mean	STD	Mean	STD	Mean	STD	*p*-value^1^
Parameter							
HbA1c [%] Mmol/mol	9.7 83	1.7	8.5 69	1.3	8.6 70	1.2	0.00009
HbA1c(relative)	100	0	89.2	14.5	89.9	10.9	0.0001
Average Daily Insulin (Units)	48.5	17.7	45.5	17.0	46.3	15.9	0.03
Average Blood Glucose (mg)	–	–	211.3	58.0	199.4	59.8	–
BP systolic	113.7	9.4	113.4	10.2	114.9	9.9	ns
BP diastolic	68.7	8.2	67.3	8.1	68.0	8.4	ns
BMI [kg/m^2^]	21.5	4.0	22.0	4.1	21.6	4.6	ns
Subjective Perception of Therapy^2^							
Satisfaction/Analog scale	56.3	12.5	70.2	13.5	75.7	12.6	0.00001
Satisfaction/Questionnaire	61.7	18.8	73.9	16.4	79.3	10.5	0.00336
Subjective Handling Ease	44.8	16.7	64.1	20.9	75.3	17.3	0.00006
Subjective Compliance	47.4	16.5	60.7	13.7	70.6	10.5	0.00003

^1^*p*-value for development from baseline to visit 2 by Wilcoxon signed rank test

^2^Analog scale 0–100 = worst to best; the questionnaire ratings (1–5) were rescaled to 0–100;

Results from baseline over visit1 to visit 2 for the secondary study group are summarized in Table 3. The pattern of results was similar to the primary study group. There was a mean decrease of HbA1c by 1.0% from baseline to visit 2. BMI increased by about one point and no changes were observed in the blood pressure. Similar to the primary group, there was a decrease in daily insulin. Subjective perception of therapy was favorable. The patient ratings of the advanced features of the system are compiled in Table 4. Ratings are largely in favor of the system.

**Table 3:** Parameters of the secondary study group over the study period (*N* = 14 Age 6–11 y)

	Baseline		Visit 1		Visit 2		
	Mean	STD	Mean	STD	Mean	STD	*p*-value^1^
Parameter							
HbA1c	8.7 72	1.9	7.7 61	0.8	7.9 63	0.9	0.09^1a^
HbA1c (relative)	100	0	91.6	16.1	93.4	16.4	0.09^1a^
Average Daily Insulin (Unit)	33.5	20.7	28.6	16.1	30.1	14.5	0.10^1a^
Average Blood Glucose (mg)	.	.	182.2	29.0	174.0	37.5	.
BP systolic	102.7	10.1	105.9	10.4	108.4	18.2	ns
BP diastolic	62.3	8.9	63.6	9.9	62.8	8.2	ns
BMI [kg/m^2^]	18.0	3.6	19.0	3.8	18.9	3.9	0.0046
Subjective Perception of Therapy^2^							
Satisfaction/Analog scale	60.0	16.2	78.6	8.6	80.7	9.2	0.0005
Satisfaction/Questionnaire	58.0	16.2	85.7	11.6	83.0	10.5	0.0005
Subjective Handling Ease	46.4	22.2	67.0	14.4	63.4	12.5	0.08^1a^
Subjective Compliance	58.6	15.5	75.0	12.0	69.1	15.1	ns

^1^*p*-value for development from baseline to visit 2 by Wilcoxon signed rank test ^1a^ trend

^2^Analog scale 0–100 = worst to best; the questionnaire ratings (1–5) were rescaled to 0–100;

**Table 4:** Rating of advanced features of the system after 6 months

	Total group *N* = 38		Primary study group *N* = 24		Secondary study group *N* = 14	
	Mean	STD	Mean	STD	Mean	STD
Satisfaction with advanced equipment	75.1	11.5	72.6	11.6	79.5	10.2
Overall functionality	77.3	16.9	74.5	17.1	82.1	16.1
Use of remote control	74.0	17.0	69.2	17.5	82.1	13.0

^1^Questionnaire ratings (1–5) were rescaled to 0–100;

Exploratory analyses (in the total study group) addressed the determinants of the decrease in HbA1c and the role of the remote control in this context. Analyses dealt primarily with the absolute change in HbA1c: HbA1c (visit 2) – HbA1c (baseline). The analyses were also repeated for the relative change in HbA1c. A first regression model to analyze determinants of the decrease in HbA1c featured the regressors baseline HbA1c, sex, age, and insulin change. The analysis revealed a major influence of baseline HbA1c (*p* < 0.0001) with further contributions of female gender (*p* = 0.03); similar results were seen for the relative change in HbA1c.

### Determinants of a high baseline HbA1c

Univariate regressions of baseline HbA1c on questionnaire variables were used to assess the most influential measures for subjective compliance. Patients who reported low subjective compliance at baseline had higher HbA1c values (*p* < 0.05); likewise, those with large improvements in subjective compliance at visit 2 had originally high HbA1c values at baseline (*p* < 0.03). This indicates that higher baseline HbA1c values were related to compliance issues under MDI.

For further analysis of the role of the remote control, the original coding for ‘use of remote control’ was recoded to a 3-point scale (remote use group 1–3). Based on the scale chosen, subjects segregated into low rater (*n* = 10), medium (*n* = 12) and high (*n* = 16) for the usefulness of the remote control. The demographic variable ‘age’ was strongly related to baseline HbA1c (*p* < 0.003) and therefore regressions of change in HbA1c included ‘remote use group’ and ‘age’ as predictors. It was found that age (*p* = 0.0174) and remote use group (*p* = 0.0497) were significant contributors to the reduction in HbA1c with results for relative change in HbA1c were *p* = 0.0248 and *p* = 0.0352, respectively. Table 5 studies the parameters for the different remote use groups. Notably there were significant trends in change of HbA1c (*p* = 0.0174 and 0.0152) and in the insulin dose change (relative *p* = 0.0394). These data underline that the use of the remote control contributed significantly to the gains in glycemic control.

**Table 5:** Change of parameters by remote control group

Variable	Remote use group1 *n* = 10		Remote use group2 *n* = 12		Remote use group3 *n* = 16		*P*-value^1^
	mean	STD	mean	STD	mean	STD	
Age [y]	12.1	3.0	16.5	6.2	11.9	3.2	ns
% Females	60		75		69		ns
Baseline HbA1c[%]	8.82	1.89	9.54	1.86	9.47	1.75	ns
Change^2^ HbA1c[%]	−0.54	1.13	−0.66	1.82	−1.49	1.35	0.0174
%Change HbA1c	95.4	9.5	95.0	17.1	85.7	10.0	0.0152
Baseline Daily Insulin (Units)	38.2	15.6	50.3	18.8	40.4	22.6	ns
Change Daily Insulin (Units)	0.22	6.03	−4.02	5.53	−5.08	10.6	0.0824^1a^
%Change Daily Insulin	102.7	23.2	91.9	10.6	85.1	13.6	0.0394

^1^Jonckheere-Terpstra non-parametric trend test; ^1a^ trend

^2^Absolute change in HbA1c: HbA1c(visit2)-HbA1c(baseline)

^3^Relative change in HbA1c in percent: 100*HbA1c(visit2)/HbA1c(baseline)

^4^Absolute change in daily insulin: insulin(visit2)-insulin(baseline)

^5^Relative change in daily insulin in percent: 100*insulin(visit2)/insulin(baseline)

### Safety

For the 3 months before baseline, there were 15 and 12 reports of DKA and hypoglycemic events in the total group, respectively. On the start of the study, one subject, 6-year-old, developed DKA in the first few weeks, and another subject reported one episode of severe hypoglycemia in period 1 (baseline to visit1). For period2 (visit 1 to visit 2), no safety events were reported. No pump malfunctions or Interruptions of remote control function were reported throughout the study.

## Discussion

The study demonstrates that switching young T1DM patients from MDI to a pump system can improve glycemic control along with a decrease in daily insulin dose. The mean decrease in HbA1c was 1.09 and 0.79% in primary and secondary study group, respectively. All aspects of patient satisfaction improved with the new system and there was a favorable safety profile. The results of the present study in principal parallel those of previous studies, but the size of the improvement in HbA1c appears remarkable.. Bayrakdar et al. reported, in matched cross-sectional study comparing MDI and CSII, a difference of 1.5% in HbA1c – however, the results were based on a limited database of 18 patients per group [12]. Most of other studies report a lesser improvement: Blackman et al. [10] reported from a large cross-sectional study *n* = 669 decreases of 0.6% in very young patients (≤ 6y); Benkhadra et al. [9] in a meta-analysis reported for the pediatric part (*n* = 543) improvements of 0.32%; similar to the decrease of 0.31% seen in *n* = 700 children by Birkebaek et al. [13]. Other studies found smaller gains when comparing MDI and pump [14–17, 22].

The larger reductions in HbA1c, observed in the present study, might have derived from an add-on effect in compliance by the advanced features of the system, in particular by use of the remote control. As the design of this observational study does not allow to explicitly separate the effects of pump and the remote control, analyses capitalizing on a special questionnaire could solidly underpin that the remote control contributed to the extent of improvement. Unfavorable baseline HbA1c values in this study were probably invoked by insufficient therapy adherence to MDI. Switching to a pump system helped to trouble shoot this issue with the remote-control system enhancing the compliance further by providing accessibility to insulin. This interpretation is backed by the fact that daily insulin decreased significantly during the study, particularly for those who gave excellent ratings regarding the utility of the remote control. Personal satisfaction, subjective ease of handling and perceived therapy compliance showed an improvement with the new system use.

While the study results are reassuring of the validity of the system use, there are limitation to the study. The uncontrolled design of the study and lack of comparison between users of the pump with or without the remote function represent limitation to the study.

## Conclusion

The use of a pump system with remote control led to therapy improvement in young T1DM patients. The ease of use of the remote insulin delivery led to favorable HbA1c reductions particularly for those with unfavorable values at baseline. Patients who rated the use of the remote control favorably, had significantly larger reductions of HbA1c and insulin dose. Patient satisfaction advanced significantly, and the safety developed favorably.

## Additional file

Additional file 1: Study Questionnaires. (DOCX 94 kb)

## Data Availability

Data is available upon request.

## Abbreviations

CSII: Continuous subcutaneous insulin infusion
DKA: Diabetes ketoacidosis
HbA1c: Haemoglobin A1c
MDI: Multiple daily injections
T1DM: Type 1 diabetes mellitus
UAE: United Arab Emirates
