# Supplementary material for: Using insulin pump with a remote-control system in young patients with diabetes improves glycemic control and enhances patient satisfaction
Source: Clin Diabetes Endocrinol. 2019 Jun 6;5:7. doi: 10.1186/s40842-019-0081-z (PMC6555038; doi:10.1186/s40842-019-0081-z)
Supplement: Supplementary file 1 — Study Questionnaires. (DOCX 94 kb) [file 40842_2019_81_MOESM1_ESM.docx]

**Questionnaire A:**

**Treatment satisfaction, regimen distress and therapy adherence (to be assessed at all visits 0, 3 months, and 6 months?)**

Living with diabetes can sometimes be tough. There may be problems and hassles and it can be difficult to accommodate the recommended therapy in daily life. Please think of the last 4 weeks and value the following statements. There is no wrong or right – please cross the respective field according to your own subjective feelings.

|  | Statement | strongly agree | agree | neutral | disagree | strongly disagree |
| --- | --- | --- | --- | --- | --- | --- |
|  | Symbols | 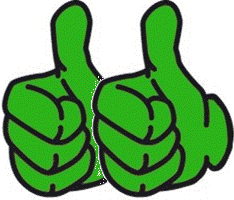  5 | 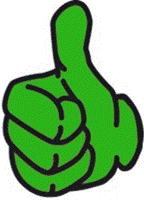  4 | 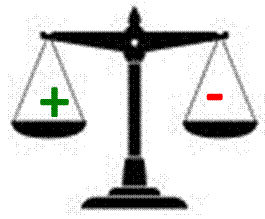  3 | 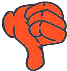  2 | 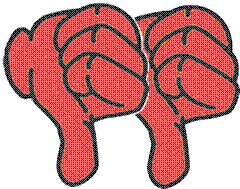  1 |
| 1 | I feel generally good with my diabetes treatment |  |  |  |  |  |
| 2 | My treatment is in general convenient |  |  |  |  |  |
| 3 | I think my blood sugars were in the target range most of the times |  |  |  |  |  |
| 4 | I would recommend this type of treatment to a friend |  |  |  |  |  |
| 5 | I do not get unusually high glucose readings |  |  |  |  |  |
| 6 | My treatment does not interfere with my activity |  |  |  |  |  |
| 7 | I am testing my blood sugars frequently enough. |  |  |  |  |  |
| 8 | I can easily receive insulin even if I am not at home |  |  |  |  |  |
| 9 | I am following the recommended frequency of blood sugar measurement and bolus determination |  |  |  |  |  |
| 10 | I infrequently need support from the diabetes team |  |  |  |  |  |
| 11 | I understand my diabetes disease sufficiently |  |  |  |  |  |
| 12 | I do not get unusually low glucose readings |  |  |  |  |  |

**Subjective health scale (to be used at all visits)**

| **Subjective Health Status** | **Analog scale** | |
| --- | --- | --- |
| To help you say how good or bad a health state is, we have drawn a scale (like a thermometer) on which the best state you can imagine is marked 100 and the worst state you can imagine is marked 0. We would like you to indicate on this scale how good or bad your own health is today, in your opinion. Please do this by crossing whichever point on the scale indicates how good or bad your health state is today. | 100  \|  90  \|  80  \|  70  \|  60  \|  50  \|  40  \|  30  \|  20  \|  10  \|  0 | **Best imaginable health state**  **Worst imaginable health state** |

**our own**

**Questionnaire B: (at 6 months)**

You have now used the new system for 3 months in the following questions, we would like know how the new system was usable in your daily therapy. Please think of the last 4 weeks and value the following statements. There is no wrong or right – please cross the respective field according to your own subjective feelings.

|  | **Statement** | **strongly agree** | **agree** | **neutral** | **disagree** | **strongly disagree** |
| --- | --- | --- | --- | --- | --- | --- |
|  | **Symbols** | **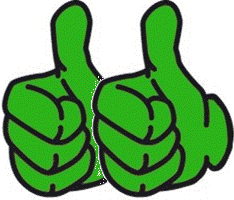** | **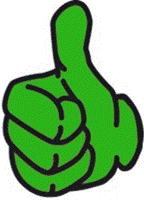** | **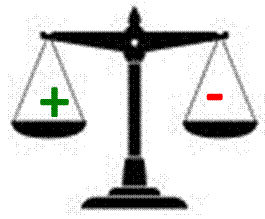** | **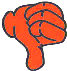** | **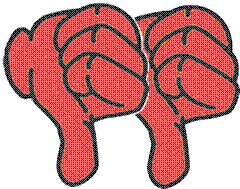** |
| 1 | The new therapy system was easy to learn and use |  |  |  |  |  |
| 2 | The remote control made it easy to apply bolus when I was not at home |  |  |  |  |  |
| 3 | The remote control worked without problems |  |  |  |  |  |
| 4 | For using the new system I needed frequently help from my parents or the diabetes team |  |  |  |  |  |
| 5 | Without the remote control the system would be equally good |  |  |  |  |  |
| 6 | Altogether I do not think that my diabetes treatment improved with combo |  |  |  |  |  |
| 7 | My friends and peers were impressed from the new technique |  |  |  |  |  |
| 8 | My parents liked the new system |  |  |  |  |  |
| 9 | The new system with remote control helped much to adhere to therapy |  |  |  |  |  |
| 10 | The bolus calculator was quick and precise in many everyday situations |  |  |  |  |  |
